# Endoscopic management of active arterial bleeding in walled-off necrosis collection

**DOI:** 10.1016/j.vgie.2025.05.013

**Published:** 2025-05-31

**Authors:** Matthew Eganhouse, Agnieszka Maniak, Kanika Garg, Thomas Wang, Neal A. Mehta, Christopher G. Chapman, Irving Waxman, Ajaypal Singh

**Affiliations:** Rush University Medical Center, Center for Interventional and Therapeutic Endoscopy, Division of Digestive Diseases and Nutrition, Chicago, Illinois, USA

## Abstract

**Background and Aims:**

Bleeding related to drainage of walled-off necrosis (WON) via a lumen-apposing metal stent (LAMS) and subsequent endoscopic necrosectomy is a known adverse event of the procedure. Typically, these bleeds are managed by interventional radiology. This case demonstrates successful endoscopic management of bleeding from a WON cavity.

**Methods:**

A 45-year-old female underwent EUS-guided cystagsastrostomy with a LAMS for a large, symptomatic WON collection. Six days after the procedure, she presented with hematemesis. Computed tomography angiography showed blood products in the WON cavity but no active arterial extravasation. The decision was made to pursue endoscopic evaluation with EGD.

**Results:**

During EGD, extensive clot extraction and removal of the LAMS allowed discovery of a pulsatile vessel in the WON cavity. This was treated with coagulation grasper forceps, ligation of vessel with hemostatic clips, and peptide matrix gel. She had no further bleeding, and WON collection resolved.

**Conclusions:**

This case portrays successful endoscopic therapy for bleeding within a WON cavity. Normally, bleeding after placement of a LAMS is managed by interventional radiology. However, endoscopic therapy should be considered when imaging does not reveal active arterial extravasation or if the bleeding is intermittent in nature.

## Case description

**Figure 1:**
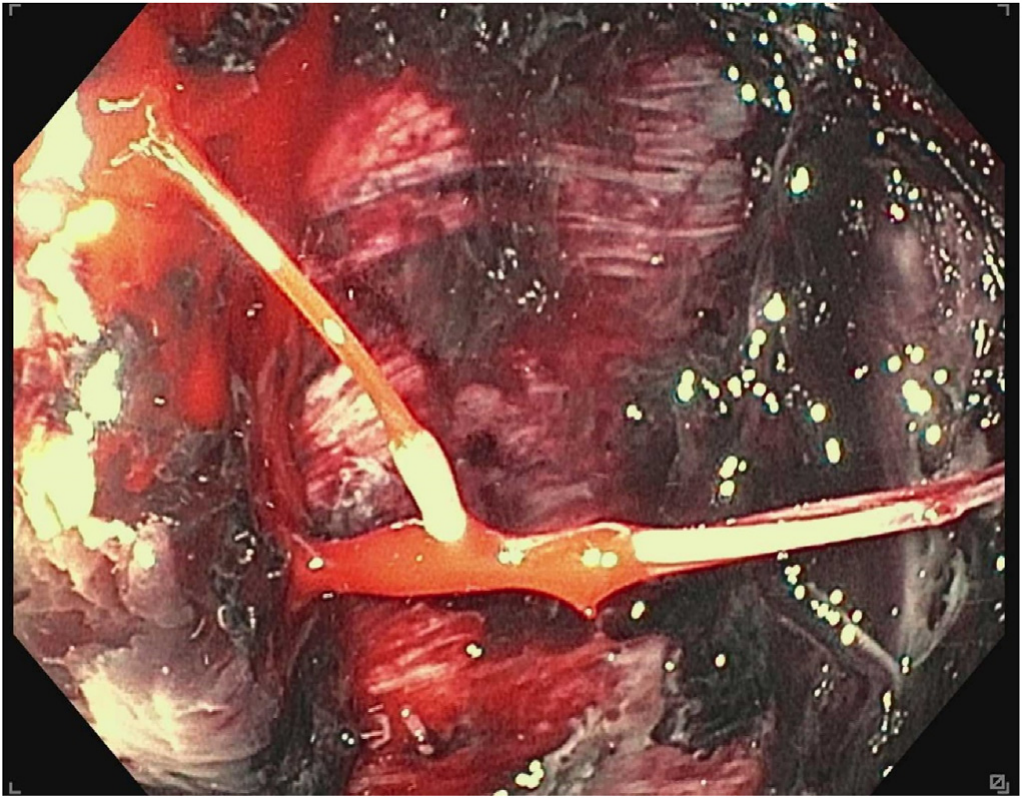
A spurting vessel in the walled-off necrosis cavity.

**Figure 2:**
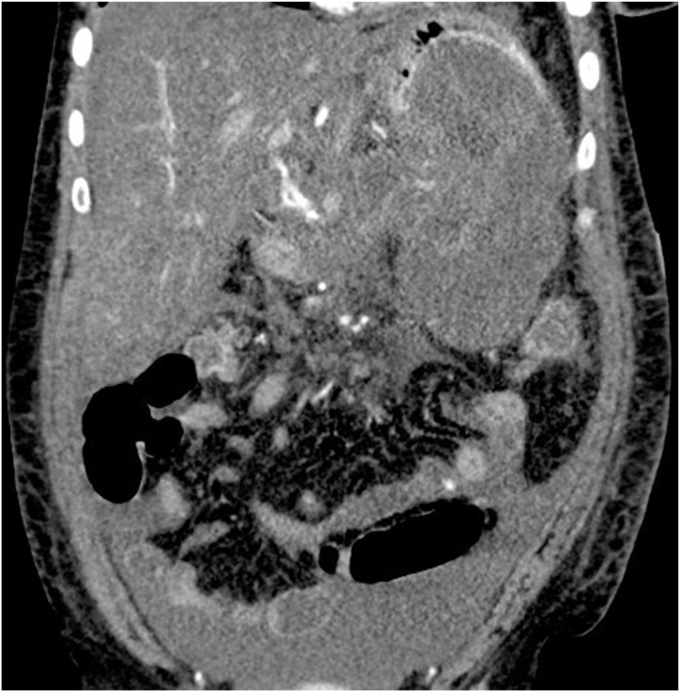
Computed tomography of the abdomen and pelvis showing necrotizing pancreatitis with walled-off collection with debris and small amount of blood products.

**Figure 3:**
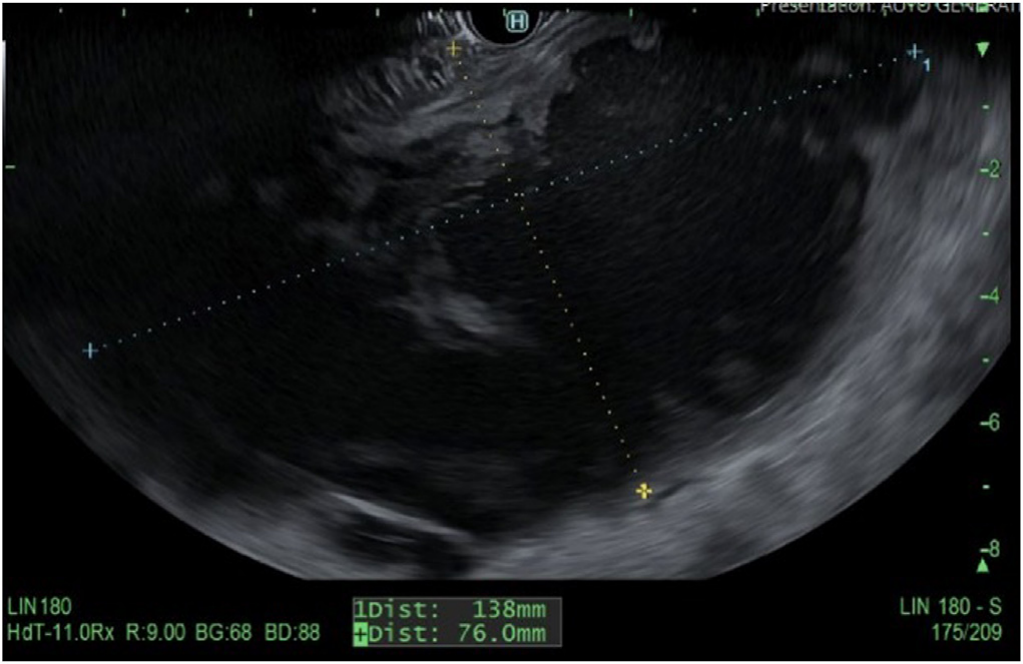
EUS showing walled-off necrosis collection.

**Figure 4:**
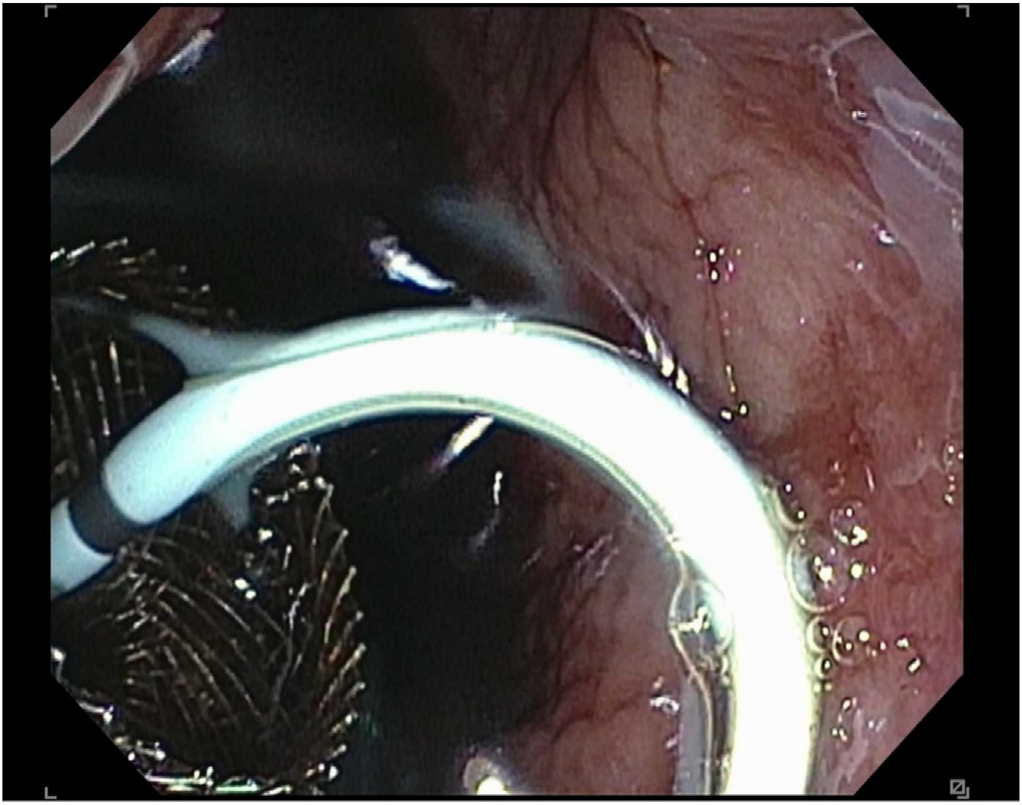
Endoscopic view after successful lumen-apposing metal stent and double-pigtail stent placement.

A 45-year-old woman presented with severe acute necrotizing pancreatitis secondary to alcohol use. She was readmitted with worsening abdominal pain 23 days later. A computed tomography (CT) scan with intravenous contrast of the abdomen and pelvis showed a 14-cm encapsulated pancreatic collection with significant mass effect on the stomach. There was no splenic artery pseudoaneurysm (Fig. 1). Because the patient was symptomatic from the collection and it appeared walled off on imaging, the patient underwent EUS-guided cystgastrostomy (Figure 2, Figure 3, and 4). A 20-mm × 10-mm lumen-apposing metal stent (LAMS) was placed using the freehand technique and a double-pigtail plastic stent was placed across the LAMS with no adverse events.

**Figure 5:**
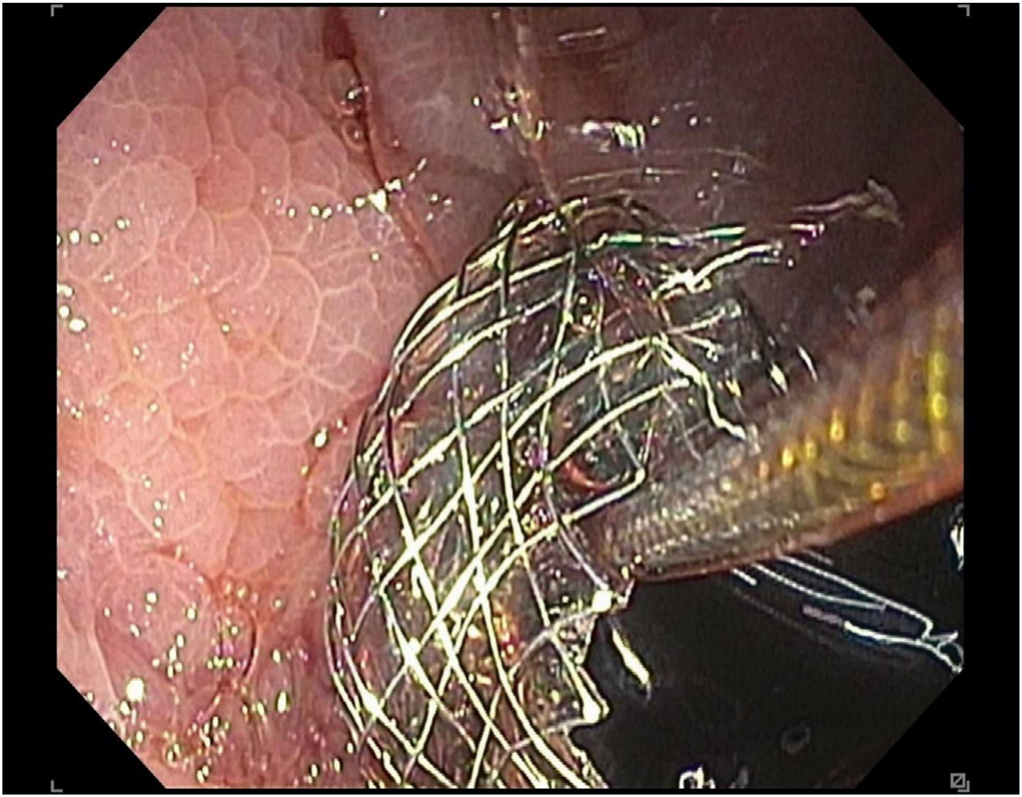
Endoscopic photo demonstrating successful placement of cystogastrostomy.

However, the patient returned 6 days later with hematemesis. On arrival, the patient was tachycardic, and hemoglobin was notably 2 g below baseline. CT angiogram (Fig. 5) was obtained twice because of concern for active bleeding, but no active extravasation or a splenic artery pseudoaneurysm was identified. As the result of continued bleeding and after multidisciplinary discussion, the patient underwent EGD.

**Figure 6:**
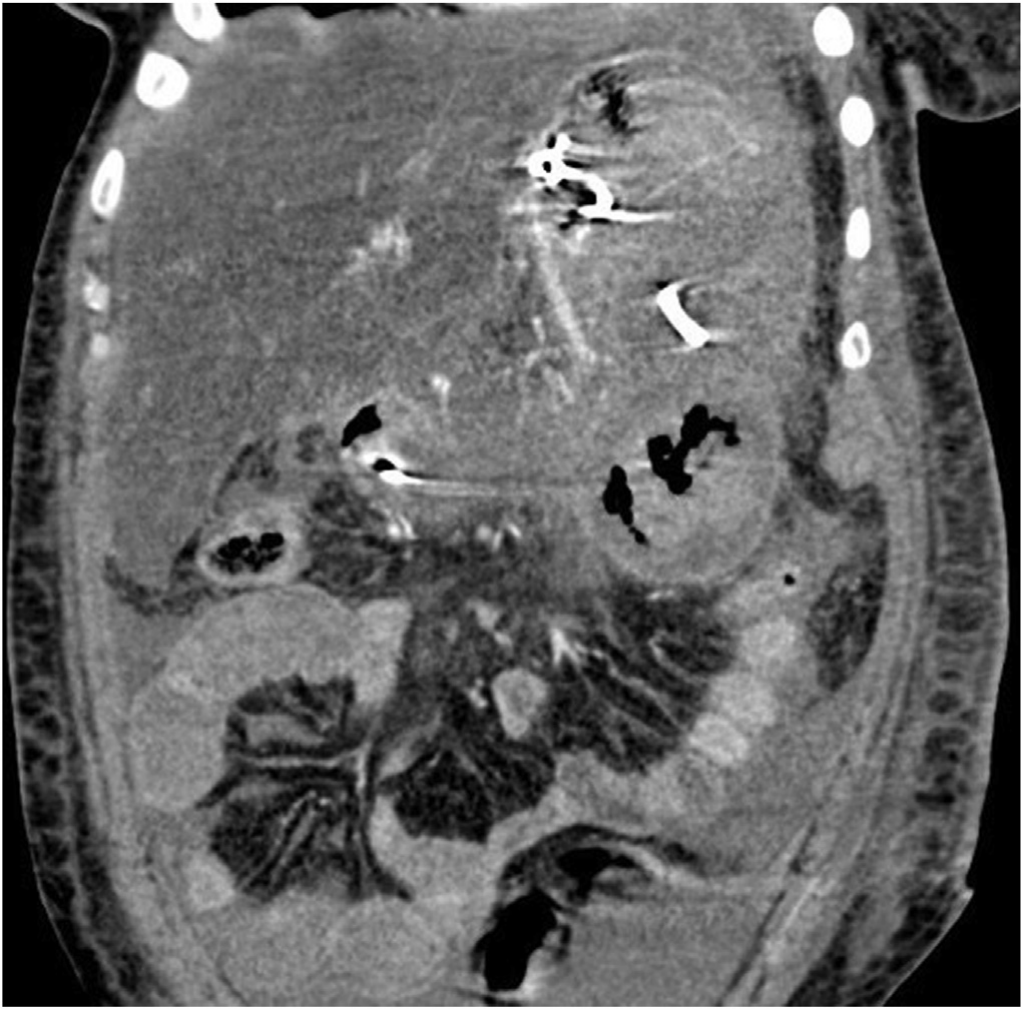
Computed tomography angiography abdomen and pelvis showing blood products in same walled-off collection.

During EGD, the previously placed plastic double-pigtail stent was removed and the single-channel therapeutic gastroscope was advanced into the walled-off necrosis (WON) cavity, which was filled with large amounts of clot and red blood (Video 1, available online at www.videogie.org). Extensive clot extraction was undertaken with a Raptor grasping device (Steris, Mentor, Ohio, USA) and snare (Boston Scientific Corporation, Marlborough, Mass, USA), requiring multiple intubations of the cyst. Initially, no active bleeding or source of bleeding was identified. To rule out bleeding related to LAMS-induced vascular injury to the opposite wall, the LAMS was removed using a grasper forceps. No bleeding was noted behind the LAMS or the opposing wall. Thus, the endoscopic necrosectomy and clot removal were continued. Active bleeding from a pulsatile vessel coursing through the collection was noted away from the cystgastrostomy site (Fig. 6).

**Figure 7:**
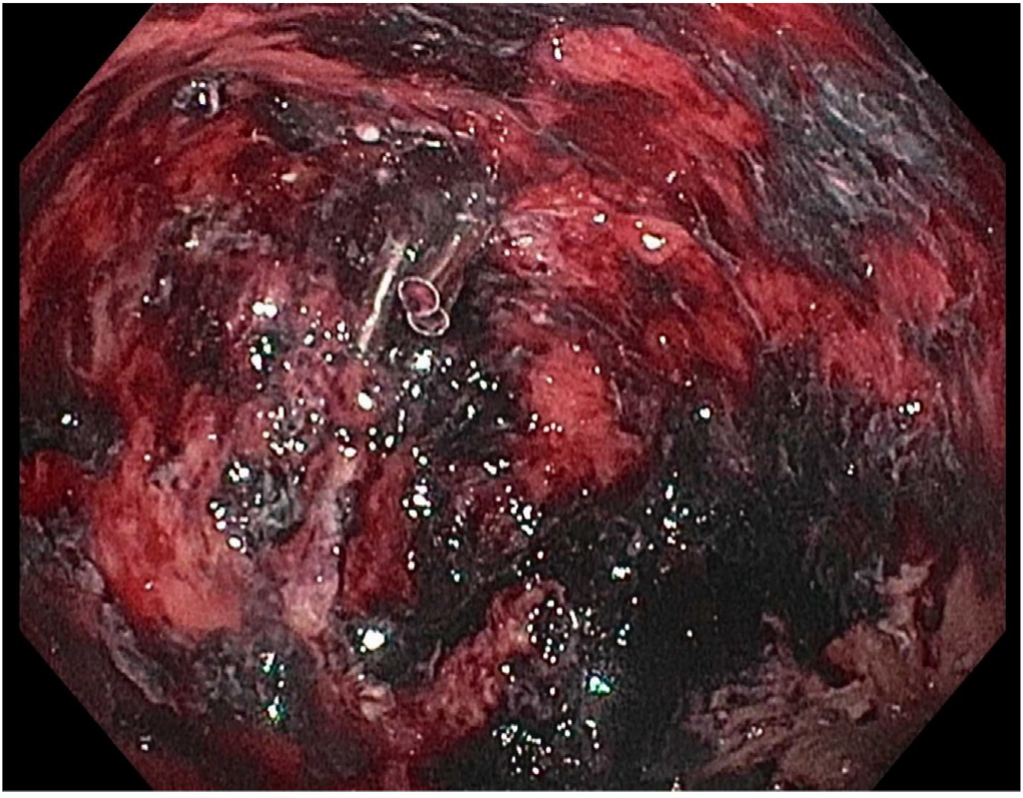
Subsequent EGD with no active bleeding.

**Figure 8:**
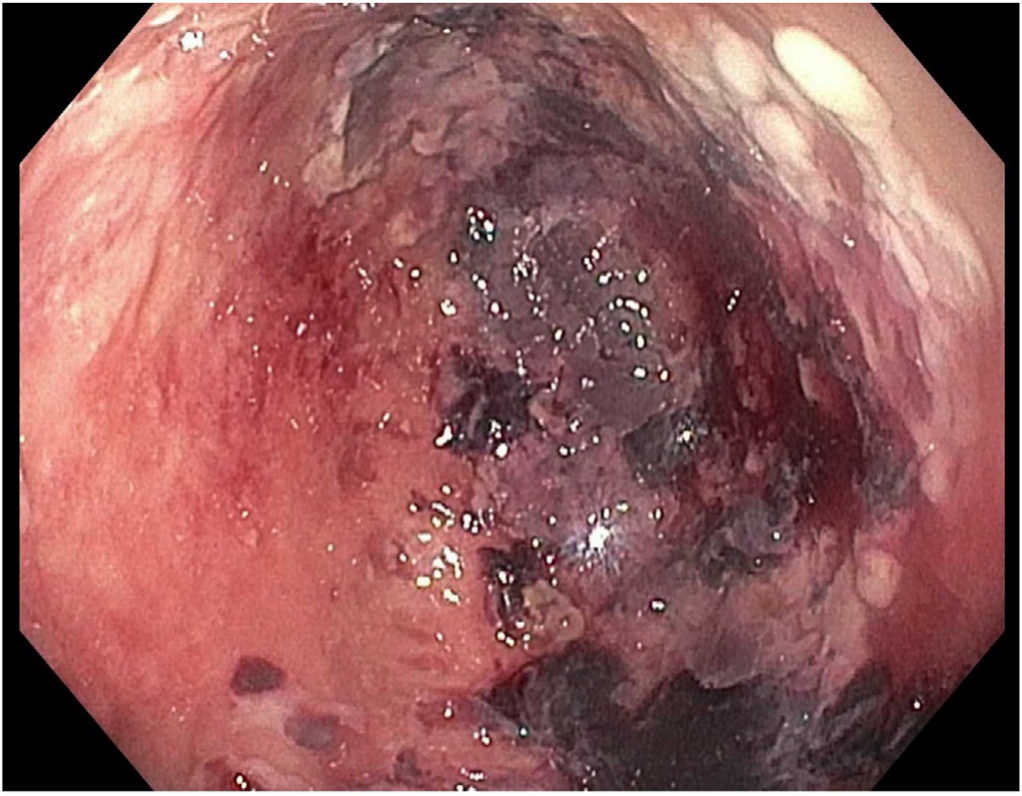
After debridement of healthy granulation tissue, small red spots were treated with bipolar thermal therapy and fibrin gel matrix.

**Figure 9:**
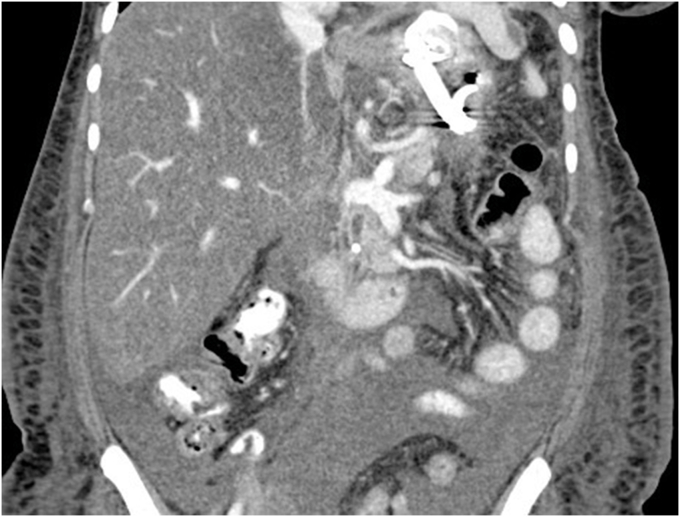
CT of abdomen and pelvis showing resolution of walled-off necrosis cavity.

Initial attempts at hemostasis using coagulation grasper forceps were only partially successful. Hemostatic clips were placed on either side of the vessel as it coursed through the WON cavity walls perpendicular to the vessel's direction to ensure appropriate hemostasis. This was followed by monopolar thermal therapy with the coagulation grasper forceps. Hemostasis was successfully achieved. A synthetic self-assembling peptide matrix gel was then applied to the bleeding site. A long guidewire was passed into the collection. Three double-pigtail stents were inserted across the cystgastrostomy tract. Repeat EGD with necrosectomy 1 week later showed no bleeding in the residual WON cavity (Figs. 7 and 8) with healthy-appearing granulation tissue on the walls. The patient had no further episodes of bleeding and had resolution of symptoms. Follow-up CT 6 weeks later showed resolution of the WON cavity (Fig. 9).

Bleeding during drainage of WON and subsequent endoscopic necrosectomy is a known adverse event and occurs as a result of direct injury of vessels during placement of a LAMS, pseudoaneurysm formation, and injury to vessels when the wall of the cavity collapses against the LAMS among other etiologies.^1^ Because of the lack of dedicated tools for necrosectomy, bleeding from mechanical injury can happen. Use of dedicated tools and/or hydrogen peroxide can increase clinical success rate.^2^ Delayed bleeding from erosion of vessels coursing through the collection is less common. Bleeding after the LAMS is usually managed by interventional radiology, particularly in cases related to splenic artery pseudoaneurysms.^3^ However, endoscopic therapy should be considered in cases in which imaging does not reveal active arterial extravasation or bleeding is intermittent in nature. If endoscopic therapy is pursued, careful inspection of the LAMS site with removal of clot to identify possible sources of bleeding is crucial. This case portrays successful endoscopic therapy for bleeding within a WON cavity using through-the-scope clips, thermal therapy, and fibrin gel matrix.

## Patient Consent

The patient consented for the use of this information for this publication.

## Disclosure

The following authors disclosed financial relationships: N.A. Mehta: Consultant for Boston Scientific and Castle Biosciences. C.G. Chapman: Consultant for Boston Scientific, Olympus, Medtronic, Steris Endoscopy, AbbVie, and Phathom Pharmaceuticals. I. Waxman: Consultant for Boston Scientific, Cook Medical, and Medtronic. A. Singh: Consultant for Boston Scientific and Creo Medical. All other authors disclosed no financial relationships.

## Figures and Tables

**Supplementary still:**
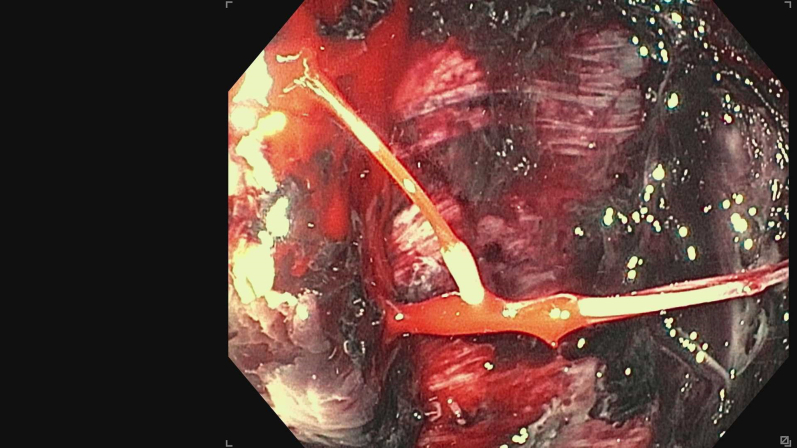


## Supplementary data

Video Endoscopic management of active arterial bleeding in walled-off necrosis collection.
